# Perceived affordability of health insurance and medical financial burdens five years in to Massachusetts health reform

**DOI:** 10.1186/s12939-015-0235-2

**Published:** 2015-10-29

**Authors:** Leah Zallman, Rachel Nardin, Assaad Sayah, Danny McCormick

**Affiliations:** Cambridge Health Alliance Department of Medicine, 1493 Cambridge St; Macht 420, Cambridge, MA 02139 USA; Institute for Community Health, Malden, MA USA; Harvard Medical School, Boston, MA USA; Cambridge Health Alliance, Cambridge, MA USA

**Keywords:** Affordability, Health Reform, Massachusetts

## Abstract

**Introduction:**

Under the Massachusetts health reform, low income residents (those with incomes below 150 % of the Federal Poverty Level [FPL]) were eligible for Medicaid and health insurance exchange-based plans with minimal cost-sharing and no premiums. Those with slightly higher incomes (150 %-300 % FPL) were eligible for exchange-based plans that required cost-sharing and premium payments.

**Methods:**

We conducted face to face surveys in four languages with a convenience sample of 976 patients seeking care at three hospital emergency departments five years after Massachusetts reform. We compared perceived affordability of insurance, financial burden, and satisfaction among low cost sharing plan recipients (recipients of Medicaid and insurance exchange-based plans with minimal cost-sharing and no premiums), high cost sharing plan recipients (recipients of exchange-based plans that required cost-sharing and premium payments) and the commercially insured.

**Results:**

We found that despite having higher incomes, higher cost-sharing plan recipients were less satisfied with their insurance plans and perceived more difficulty affording their insurance than those with low cost-sharing plans. Higher cost-sharing plan recipients also reported more difficulty affording medical and non-medical health care as well as insurance premiums than those with commercial insurance. In contrast, patients with low cost-sharing public plans reported higher plan satisfaction and less financial concern than the commercially insured.

**Conclusions:**

Policy makers with responsibility for the benefit design of public insurance available under health care reforms in the U.S. should calibrate cost-sharing to income level so as to minimize difficulty affording care and financial burdens.

**Electronic supplementary material:**

The online version of this article (doi:10.1186/s12939-015-0235-2) contains supplementary material, which is available to authorized users.

## Introduction

High rates of un-insurance [1] and financial burdens caused by declining affordability of medical care [2, 3] were the primary impetuses for both the Affordable Care Act (ACA) and Massachusetts’ health care reform, upon which the ACA was closely modeled. The ACA dramatically expands health insurance coverage to low and moderate income individuals through a Medicaid expansion and new health insurance exchange-based publicly subsidized plans [4]. Medicaid is a government insurance plan available for those with very low incomes (below 133 % of the Federal Poverty Level [FPL]). It provides comprehensive coverage for medical and mental health needs and has minimal cost-sharing requirements and no premiums. Those with low to moderate incomes (between 133 % and 400 % FPL) can obtain subsidized insurance coverage on health insurance exchanges; these subsidized plans require premiums and co-payments for most medical services. Premiums and co-payments could reduce government fiscal pressures associated with the expansion by sharing the financial burden with enrollees. However, if cost-sharing is high relative to income, enrollees could still face difficulty affording care [4, 5], forgo essential care [6] or experience financial burdens associated with receiving care [7].

A recent national survey found that users of ACA insurance exchanges, which opened in October of 2013, reported difficulty finding affordable plans [8]. No previous research has examined whether perceived affordability of health care or financial burdens varies among enrollees in the low versus higher cost-sharing public plans that formed the backbone of the ACA insurance expansion. The Massachusetts health reform, which shared most key features of the ACA, was fully implemented by 2008 and provides an opportunity to examine these questions in the setting of a comparable but more mature reform.

As with the ACA, under the MA reform law [9], cost-sharing differed among commercial (private) and public plans available to Massachusetts residents. Sixteen percent of the newly insured under the Massachusetts reform obtained commercial or employer sponsored insurances; [10] these insurances varied in their cost-sharing and coverage features but nearly all had at least some cost-sharing. 84 % gained Medicaid or a publicly subsidized insurance, Commonwealth Care (CWC), through the state insurance exchange [10]. Residents received one of three types of CWC, each with different cost-sharing and income eligibility requirements (Additional file 1: Table S1). The features of CWC Type 1 (available to residents with incomes <150 % FPL) were nearly identical to Medicaid and included comprehensive covered services, and no deductibles, coinsurance or copayments other than for medications (up to $3.65 per medication per month). CWC Types 2 and 3 (available to residents with income 150- 300 % FPL) included the same comprehensive covered services (except that they excluded dental coverage). These plans required premium payments and co-payments for most medical services, comparable to co-payments seen in commercial plans. Thus, those with the lowest incomes received low cost-sharing public plans (CSP) (Medicaid and CWC Type 1) while those with higher incomes received higher CSP (CWC Types 2 and 3).

Because it will be several years before the ACA reform has matured enough to provide data, we turn to the MA health reform five years after its full implementation to understand elements of affordability that have relevance to the ACA. Specifically, understanding the role of calibration of cost-sharing to income in affording care under MA health reform may help refine the implementation of the ACA in order to improve affordability and reduce disparities under the ACA. We sought to describe and compare the perceived affordability of insurance, perceived affordability of care, financial concerns of health care costs and satisfaction with insurance among Massachusetts residents with low and high CSP and commercial plans. We did so at a time by which Massachusetts had gained substantial experience developing and revising features of the reform and in a population with experience using the health care system.

## Methods

### Study design and setting

We conducted face-to-face surveys of a convenience sample of 976 patients presenting to three Emergency Departments (EDs) at MA’s second largest safety net hospital system, located in three communities in eastern Massachusetts (Everett, Somerville and Cambridge, MA), between August 2013 and January 2014. Although the ACA rollout began in January 2014, Medicaid and CWC products and processes for obtaining them remained essentially unchanged during the initial ACA rollout.

We surveyed patients with commercial insurance, Medicaid and CWC plans. Insurance type was determined by electronic querying of a continuously updated insurance database maintained by a consortium of all Massachusetts health insurers, including public payers [11, 12]. This database allows real-time determination of insurance type and status; as it relies on information reported directly from insurers, determination of insurance type is highly accurate. We combined patients with CWC Types 2 and 3, which we refer to as ‘high CSP’ as they both required monthly premium payments and had moderate (but different) co-payments for most services such as medications ($10 to $50), primary care visits ($10 to 15), and ED visits ($50 to $100) (Additional file 1: Table S1). We combined patients with Medicaid and CWC Type 1, which we refer to as ‘low CSP’ as neither required premium payments nor cost-sharing other than medication copayments of $3.65 or less (Additional file 1: Table S1) and both covered a nearly identical set of services. We also examined commercially insured individuals, a group for whom we were unable to identify cost sharing requirements.

The Cambridge Health Alliance Institutional Review Board approved the study protocol.

### Study subjects

We included patients aged 18–64 years who self reported that they spoke one of four languages (English, Spanish, Portuguese, or Haitian Creole) and had an Emergency Severity Index (ESI) of 2–5 (excludes the most severely ill, ESI of 1). This score is a validated ED triage algorithm that stratifies patients into five groups from 1 (most urgent) to 5 (least urgent) [13]. We excluded patients with altered mental status, inability to speak, and those who had learned of a change in insurance on the day of the interview. Patients with more than one type of insurance were excluded to allow us to isolate the impact of each insurance type. Our sample was unselected with regard to current or past medical conditions and thus included persons with chronic diseases and those without.

### Study recruitment and data collection

Trained research assistants in the EDs recruited study subjects and verbally administered the survey. Research assistants identified potential participants and approached patients in their examination rooms after confirming with the clinical care team that recruitment would not impact clinical care. Research assistants conducted detailed verbal informed consent with potential study participants in which potential harms and benefits of participation were discussed. For patients whose primary language was Spanish, Portuguese or Haitian Creole, an interpreter or bilingual research assistant was used for study consent and survey administration. Participants received $10 gift cards. Interviews were conducted 9:00 am-11:00 pm seven days per week.

### Survey development

We developed a survey instrument incorporating mainly questions from previous studies [7, 12, 14, 15]. We pilot tested the instrument with 50 patients and reviewed it with colleagues who have expertice in health policy and financing. Similar to these studies [12, 14], we inquired about perceived affordability of care by asking if participants had delayed or forgone care in the past 12 months due to cost (or since obtaining their current insurance if obtained less than 12 months prior). We considered any patient affirmatively answering for any of the following services as having perceived affordability barrier to medical care: preventive care screening, specialist care, mental health care, tests, home services, regular doctor visits, prescription medications, or physical therapy. We considered an affirmative answer to delaying or forgoing dental or vision care as indicating a perceived affordability related barrier to non-medical health care.

We assessed perceived affordability of insurance by asking about agreement with the statement “your insurance plan is affordable to you”; we also asked “Are you worried that you will not be able to pay your premium”, with “yes” and “no” response categories. We assessed satisfaction with insurance by asking respondents to rate their level of satisfaction on a four point scale from ‘very satisfied’ to ‘not satisfied at all’.

We assessed financial concerns with questions about financial burden and concerns about paying for the current ED visit. We asked participants whether they had had to set up a payment plan with a hospital or doctor's office, had had problems paying or had been unable to pay medical bills, or had trouble paying for other basic needs such as food, heat, and rent because of medical costs. As prior research has shown [7], we considered respondents to have a financial burden if they affirmatively answered any of these three questions.

Trained medical interpreters translated the survey into Spanish, Portuguese and Haitian Creole.

### Statistical analysis

To assess for potential non-response bias, we compared the mean ages, gender and distribution of ESI scores between respondents and non-respondents.

For each outcome we calculated the percentage of respondents’ answers, according to whether they were currently enrolled in a low or high CSP or a commercial plan. We used chi square tests to perform pairwise comparisons of these percentages.

Because our objective was to describe and compare the actual experiences and perceptions of patients, for our primary analyses we present unadjusted percentages. However, in order to understand the degree to which differences in patient characteristics might influence responses, we performed multivariate logistic regression analyses that controlled for race/ethnicity, language of survey (English vs. non-English), gender, education (completed high school or higher vs. less than high school), and chronic medical condition (any vs. none) [7, 16–18]. We excluded income from the models because of that variable’s collinearity with insurance type. We initially explored the relationship between the outcomes of satisfaction and perceived affordability, chronic disease and racial and ethnic background, language and income. We found no association between chronic disease and income but did find that chronic disease differed by racial and ethnic background and language. In order to better understand if these relationships impacted our outcomes, we tested whether including an interaction term between chronic disease and racial and ethnic background and one for chronic disease and language in our models affected the relationship between cost sharing group and the outcomes. Because inclusion of these interaction terms did not affect this relationship, we did not include an interaction term in our final models.

All analyses were performed using SAS software version 9.3 (SAS Institute, Cary, North Carolina).

## Results

Overall 1,188 patients were invited to participate and 976 (82 %) agreed. There were no differences in age or gender between respondents and non-respondents. Non-respondents were more likely to have ESI scores of 2–3 (vs. 4–5), indicating higher medical acuity, as compared to respondents (64 % vs 54 % respectively, *p* = 0.0124).

5 % had high CSP (as compared to a state average of 1 %) [10], 63 % had low CSP, and 32 % were commercially insured. Table 1 demonstrates the demographic characteristics of the sample.

**Table 1:** Demographics

	Full Sample (*N* = 976)	High Cost Sharing Public, *N* = 52	Low Cost Sharing Public, *N* = 614	Commercial *N*=310	P value
		(5 %)	(63 %)	(32 %)	
	%	%	%	%	
Male	38	31	33	50	<0.0001
Foreign-born	42	69	45	32	<0.0001
Education ≥ high school	87	86	82	96	<0.0001
Race					<0.0001
Black, non-Hispanic	16	18	17	14	
White, non-Hispanic	50	30	45	62	
Hispanic	28	46	31	18	
Other	6	6	6	6	
Age					
18−30	37	19	37	40	0.0036
31−45	36	33	37	36	
46−65	27	48	26	24	
Annual Income <$20,000	61	48	78	31	<0.0001
Employed	63	84	47	90	<0.0001
Emergency Severity Index of 4 or 5	47	48	44	47	0.7803
Any Rx since on plan	77	77	80	69	0.0023
Number of Doctors Visits in past year	19	14	18	23	0.1088
Hospitalization past year	23	18	28	16	0.0002
Excellent or very good Health status	39	51	31	52	<0.0001

### Perceived affordability barriers to care (delayed or forgone care due to cost)

**Fig. 1:**
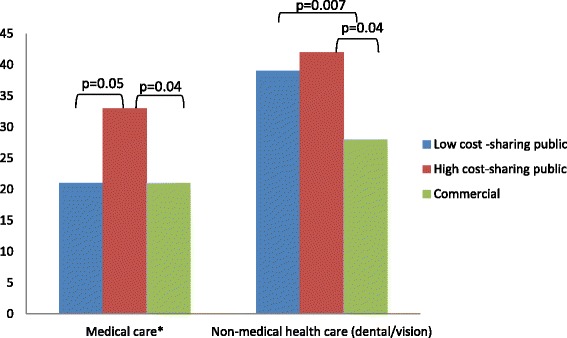
Perceived affordability barriers to care: delayed or forgone care due to cost. *Includes medications, regular and specialist doctor visits, mental or emotional care, preventive care, tests and physical therapy. ^ p values are not displayed for non-significant comparisons

Overall, 21 % indicated having perceived affordability barriers to medical care (Fig. 1) and 36 % reported having perceived affordability barriers to non-medical health care (vision/dental care). As compared to the commercially insured, higher proportions of patients with high CSP reported perceived affordability barriers to medical care (21 % vs. 33 %, respectively, *p* = 0.04) and non-medical health care (28 % vs. 42 %, respectively, *p* = 0.04). A higher proportion of high CSP recipients reported perceived affordability barriers to medical care as compared to patients with low CSP, though this was of borderline significance (33 % vs. 21 %, respectively, *p* = 0.05). A higher proportion of patients with low CSP reported perceived affordability barriers to non-medical health care than the commercially insured (39 % vs 28 %, *p* = 0.007). There were no differences in perceived affordability barriers to medical care between low cost-sharing public plan recipients and the commercially insured.

### Satisfaction with and perceived affordability of insurance

**Fig. 2:**
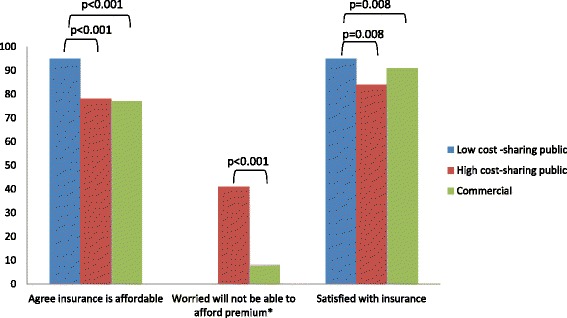
Satisfaction with and perceived affordability of insurance^. ^ p values are not displayed for non-significant comparisons. *Not applicable to low cost-sharing plans which have no premiums

Overall 88 % perceived their insurance as affordable and 93 % were satisfied with their insurance however, these proportions varied by insurance group (Fig. 2). Low CSP recipients were more likely to perceive their insurance was affordable and to be satisfied than both high CSP patients and the commercially insured. High CSP recipients were equally likely to report their insurance was affordable (78 % vs 77 %; *p* = 0.95) but were more likely to report being worried about affording their insurance premiums than were commercially insured (41 % vs 8 %; *p* <0.001). There were no differences in satisfaction between high CSP and the commercially insured.

### Financial concerns

**Fig. 3:**
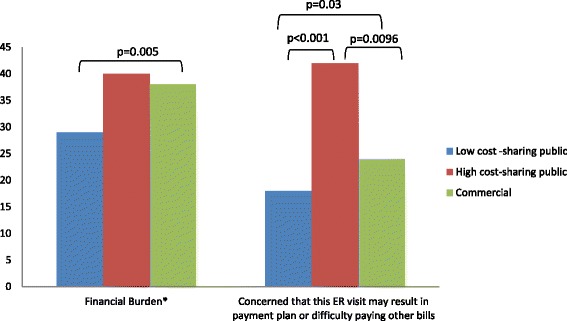
Financial Concerns^. *defined as affirmative answer to any of the following: (1) setting up a payment plan with a hospital or doctor’s office; (2) problems paying or unpaid medical bills for their medical care or the care of anyone insured under their plan; (3) trouble paying for other basic needs such as food, heat, and rent because of medical costs. ^ p values are not displayed for non-significant comparisons

Overall, 33 % reported experiencing financial burdens and 22 % reported being concerned about paying for the current ED visit (Fig. 3). As compared to the commercially insured, fewer patients with low CSP reported financial burdens (38 % vs 29 %; *p* = 0.005) and concern about affording the current ED visit (18 % vs 24 %; *p* = 0.03, respectively). Low CSP recipients were less likely to be concerned about the financial consequences of the current ED visit as compared to high CSP recipients (18 % vs 42 %; *p* < 0.001). There were no differences in the proportion reporting financial burdens between high CSP recipients and the commercially insured but more of those with high CSP had concerns about the financial consequences of the current ED visit than the commercially insured (42 % vs 24 %, *p* = 0.0096).

### Multivariate adjusted analyses: impact of patient characteristics

Adjustment for patient characteristics did not significantly alter comparisons between low and high CSP recipients or the commercially insured (Additional file 1: Tables S2–S4). Adjustment for patient characteristics modestly reduced differences between high CSP recipients and the commercially insured for three outcomes: perceived affordability barriers to medical care and non-medical health care, and being worried about affording their insurance premiums.

## Discussion

This study is the first of which we are aware to compare perceived health plan affordability, financial burdens and plan satisfaction among individuals with low and high CSP (Medicaid and health exchange-based subsidized plans) that were the core of the Massachusetts reform and are analogous to plans under the ACA, or to compare these public plan recipients with commercially insured individuals. Among patients receiving care in EDs of a large safety net health care system five years after full implementation of Massachusetts health reform, we found that high CSP recipients were more likely to report perceived affordability barriers to medical care than those with low cost-sharing public plans (borderline significance) or commercial insurance and less likely to perceive that their insurance was affordable than those with low cost-sharing public plans. High CSP recipients were more likely to report concern about the financial consequences of their ED visit; 40 % reported experiencing financial burdens but this was not significantly more than the other insurance groups. High CSP recipients were also more likely to be worried about affording their premiums compared with those with commercial insurance. In addition, we found that for most of these outcomes, low cost-sharing public plan recipients fared better than those with commercial insurance. Lastly, satisfaction with insurance exceeded 90 % for low cost-sharing plan recipients and the commercially insured and was significantly higher for both than for those with high CSP.

Low income individuals have difficulty affording basic needs including medical care [19, 20]. It is therefore notable that despite their low incomes, low CSP recipients had lower proportions reporting financial barriers to medical care and medically caused financial burdens, and that 95 % felt their insurance was affordable and were satisfied with their plan. This suggests that the public plans offered though the Massachusetts reform that cover a wide range of services and require no premiums and small copayments allowed the recipients to afford needed care and avoid financial hardship associated with medical care.

High CSP recipients had somewhat higher incomes (ranging between $11,490 and $34,470) and plan features that were much the same as low CSP except in required copayments and premiums; high CSP monthly premiums ranged between $3 to $182 and copayments for medications, for example, ranged from $10 to $50 (Additional file 1: Table S1). Our finding that high CSP were more likely to report financial barriers to medical care and less likely to perceive their insurance as affordable compared with low CSP recipients may suggest that copayments for high CSP are high enough in relation to income that difficulties with perceived affordability persist. The finding that 41 % of high CSP recipients were worried about affording their insurance premium (compared with 8 % of the commercially insured) may similarly suggest that, in relation to income, premiums levels were high enough to cause some psychological distress.

An alternative explanation is that differences in population characteristics other than income account for differences in satisfaction and perceived affordability. The fact that multivariate analyses did not significantly alter our findings suggests that these differences do not play a large role in explaining our results. In particular, we explored the relationship between the outcomes of satisfaction and perceived affordability, chronic disease and racial and ethnic background, language and income. Our finding that inclusion of interaction terms between chronic disease and (1) racial and ethnic background and (2) language did not significantly alter our findings suggests that these relationships do not play a large role in explaining our results.

This study was designed to gain an in-depth understanding in a convenience sample of patients seeking emergency care at safety net hospitals. It was not intended to be representative of the general population in MA. Patients with publicly subsidized forms of insurance are more likely to seek care in safety net institutions, so this research design allowed us to locate such patients efficiently and to focus our investigation on persons utilizing their health insurance. By recruiting respondents from the EDs, we were able to verify insurance types with nearly 100 % accuracy, thus reducing the high error rates introduced by asking respondents to identify their insurance type [21]. In contrast to nearly all previous studies of MA health reform, this allowed us to determine the cost-sharing design of participants with publicly subsidized insurances and compare these outcomes according to the design among public plans that are the core of the reform.

We excluded persons with multiple insurance types and thus our results do not reflect the experiences among such individuals. We also excluded the sickest patients; it is unlikely that the most acutely ill would have reported different experiences but this possibility cannot be excluded. Our power to detect statistically significant differences between high CSP participants and other groups was likely limited by the small sample of high CSP participants. Perceived affordability of insurance was assessed according to respondents’ subjective perceptions. It is possible that using an objective definition would have yielded different responses. It is important to note that our sampling frame resulted in a substantially higher response rate than population-based surveys, decreasing the chance of non-response bias. Our survey was conducted in 4 languages, many of which were excluded from most prior surveys which have been conducted only in English and Spanish.

## Conclusions

Our findings suggest that perceived affordability of care and insurance, and satisfaction with insurance differ among individuals receiving insurance types with differing cost-sharing requirements that formed the backbone of MA health reform, at least among our convenience sample of patients. Although we do not specifically elicit the reasons for these differences, one explanation that is potentially amenable to policy modifications is that cost-sharing requirements for some insurance types may not have been calibrated to income in such a way as to allow care to be equally affordable.

To the extent that our findings apply to the cost-sharing imposed under the ACA, this study identifies an important policy issue. Under the ACA, persons with incomes under 150 % FPL have similar cost-sharing as among the low cost-sharing public plan group in our study [22]. For those with incomes above 150 % FPL, cost-sharing under the ACA is higher than studied here [22]. Future investigations on the impact of ACA cost-sharing could determine whether our findings are applicable to the higher cost-sharing for those with incomes above 150 % FPL under the ACA.

## Additional file

Additional file 1.
**Appendix Tables.** (DOCX 23 kb)
