# Supplementary material for: Perceived affordability of health insurance and medical financial burdens five years in to Massachusetts health reform
Source: Int J Equity Health. 2015 Oct 29;14:113. doi: 10.1186/s12939-015-0235-2 (PMC4625927; doi:10.1186/s12939-015-0235-2)
Supplement: Additional file 1. — Appendix Tables. (DOCX 23 kb) [file 12939_2015_235_MOESM1_ESM.docx]

**Appendix Tables**

**Appendix Table 1.** **Cost Sharing and Health Benefits in Massachusetts Public Health Insurance Plans in 2013**

| Cost Sharing | Public Insurance Plan | | | |
| --- | --- | --- | --- | --- |
|  | Medicaid ^*^ | Commonwealth Care Plans^†^ | | |
|  |  | Type 1 | Type 2 | Type 3 |
| Copays (dollars) |  |  |  |  |
| Outpatient visit |  |  |  |  |
| Primary Care provider | 0 | 0 | 10 | 15 |
| Specialist | 0 | 0 | 18 | 22 |
| Inpatient care | 3 | 0 | 50 | 250 |
| Emergency care | 0 | 0 | 50 | 100 |
| Prescription drugs (30 days) |  |  |  |  |
| Generic drug | 1-3.65 | 1-3.65 | 10 | 12.50 |
| Drug on preferred list | 3.65 | 3.65 | 20 | 25 |
| Drug not on preferred list | NC | 3.65 | 40 | 50 |
| Imaging (CT, MRI, PET) |  | 0 | 30 | 60 |
| Vision (eye exam) | 0 | 0 | 10 | 20 |
| Rehabilitation services | 0 | 0 |  |  |
| Inpatient (Rehab Hospital) | 3 | 0 | 50 | 250 |
| Outpatient Visit | 0 | 0 | 10 | 20 |
| Maternity and family planning | 0 | 0 | 0 | 0 |
| Dental | 0 | 0 | NC | NC |
| Maximum copay for prescription | 250 | 250 | 400 | 650 |
| Maximum copay excluding prescriptions | 36 | 0 | 600 | 1200 |
| Premium (dollars/month) | 0^‡^ | 0 | 3-81 | 118-182 |
| Deductible (dollars) | 0^§^ | 0 | 0 | 0 |

Source: HealthConnector Website [1] and [Mass.Gov](http://Mass.Gov) website [2]

NC=Service not covered

^*^ In Massachusetts there are seven separate Medicaid plans for those under 65 years of age. We report the covered benefits and cost-sharing for MassHealth Standard, MassHealth Basic, MassHealth Family Assistance, MassHealth CommonHealth and MassHealth Essential. MassHealth Limited and MassHealth Prenatal have very limited covered benefits.

| **Appendix Table 2. Affordability-related barriers to care: Delayed or Foregone Care Due to Cost^** | | | | | | | |
| --- | --- | --- | --- | --- | --- | --- | --- |
| \|  \|  \|  \|  \|  \|  \| \| --- \| --- \| --- \| --- \| --- \| --- \| | Low cost sharing plan vs commercially insured | | High cost sharing plan vs commercially insured | | High cost sharing plan vs  Low cost sharing plan | |  |
|  | Unadjusted  OR (CI) | Adjusted*  OR (CI) | Unadjusted  OR (CI) | Adjusted*  OR (CI) | Unadjusted  OR (CI) | Adjusted*  OR (CI) |  |
| Medical care* | 1.06 (0.76-1.49) | 0.93 (0.65-1.35) | **1.94 (1.02-3.70)** | **1.66 (0.85-3.26)** | 1.83 (0.99-3.37) | 1.78 (0.95-3.34) |  |
| Non-Medical care^&^ | 1.67 (1.24-2.24) | 1.46 (1.05-2.02) | **1.88 (1.03-3.44)** | **1.34 (0.71-2.55)** | 1.13 (0.64-2.00) | 0.92 (0.51-1.68) |  |

^bolded items are those for which adjustment for demographic differences changes the significance of the findings

*Adjusted for race/ethnicity, gender, language of survey, education, and chronic medical condition

| **Appendix Table 3. Satisfaction with and perceived affordability of insurance^** | | | | | | |
| --- | --- | --- | --- | --- | --- | --- |
|  | Low cost sharing plan vs commercially insured | | High cost sharing plan vs commercially insured | | High cost sharing plan vs  Low cost sharing plan | |
|  | Unadjusted  OR (CI) | Adjusted*  OR (CI) | Unadjusted  OR (CI) | Adjusted*  OR (CI) | Unadjusted  OR (CI) | Adjusted*  OR (CI) |
| Perceived affordability of insurance |  |  |  |  |  |  |
| Disagree insurance is affordable | 5.01  (3.14-7.99) | 7.19  (4.15-12.45) | 0.98  (0.47-2.00) | 1.4  (0.60-3.20) | 0.20  (0.10-0.40) | 0.20  (0.09-0.46) |
| Worried will not be able to afford premium | n/a | n/a | **4.29**  **(1.96-9.60)** | **2.20**  **(0.80-6.00)** | n/a | n/a |
| Dissatisfaction with Insurance | 2.09  (1.20-3.66) | 2.56  (1.4-4.7) | 0.5  (0.3-1.3) | 0.7  (0.3-1.7) | 0.23  (0.11-0.60) | 0.39  (0.2-0.7) |

^bolded items are those for which adjustment for demographic differences changes the significance of the findings

*Adjusted for race/ethnicity, gender, language of survey, education, and chronic medical condition

| **Appendix Table 4. Financial Concerns** ^ | | | | | | |
| --- | --- | --- | --- | --- | --- | --- |
|  | Low cost sharing plan vs commercially insured | | High cost sharing plan vs commercially insured | | High cost sharing plan vs  Low cost sharing plan | |
|  | Unadjusted  OR (CI) | Adjusted*  OR (CI) | Unadjusted  OR (CI) | Adjusted*  OR (CI) | Unadjusted  OR (CI) | Adjusted*  OR (CI) |
| Financial burden | 0.66  (0.50-0.90) | 0.58  (0.42-0.80) | 1.09  (0.60-1.98) | 0.92  (0.49-1.72) | 1.65  (0.92-2.94) | 1.58  (0.87-2.88) |
| Concerned that this emergency room visit may result in payment plan or difficulty paying for other bills | 0.68  (0.48-0.97) | 0.59  (0.40-0.86) | 2.24  (1.21-4.18) | 1.93  (1.01-3.72) | 3.29  (1.80-6.00) | 3.30  (1.77-6.15) |

^bolded items are those for which adjustment for demographic differences changes the significance of the findings

*Adjusted for race/ethnicity, gender, language of survey, education, and chronic medical condition

**References**

1. HealthConnector. [cited February 21, 2014]; Available from: https://[www.mahealthconnector.info/portal/site/connector/menuitem.fefd6886b932e8535734db47e6468a0c/?fiShown=default](http://www.mahealthconnector.info/portal/site/connector/menuitem.fefd6886b932e8535734db47e6468a0c/?fiShown=default).

2. Commonwealth of Massachusetts. *MassHealth Provider Manual Series.* . [cited 1/28/2015]; Available from: <http://www.mass.gov/eohhs/gov/laws-regs/masshealth/provider-library/provider-manual/>
